# Quantifying the dosimetric impact of online daily adaptation for MR-guided RT in cervical cancer

**DOI:** 10.2340/1651-226X.2025.42898

**Published:** 2025-05-19

**Authors:** Amerah Alshamrani, Robert Chuter, Marianne Aznar, Peter Hoskin, Claire Nelder, Ananya Choudhury, Lisa Barraclough, Cynthia L. Eccles

**Affiliations:** a Division of Cancer Sciences, School of Medical Sciences, Faculty of Biology, Medicine and Health, The University of Manchester, Manchester, United Kingdom; b Department of Radiological Sciences, College of Health and Rehabilitation Sciences, Princess Nourah bint Abdulrahman University, Riyadh, Kingdom of Saudi Arabia; c Department of Clinical Oncology, Christie NHS Foundation Trust, Manchester, United Kingdom

**Keywords:** Cervical cancer, MRgART, Online adaptation, inter-intrafraction dosimetric impact

## Abstract

**Purpose:**

This study assessed the inter- and intra-fractional dosimetric impact of MR-Linac-based adaptive radiotherapy for cervical cancer (CC).

**Methods:**

A retrospective analysis of five node-negative, locally advanced cervical cancer patients treated under the MOMENTUM study (NCT04075305) using adapt-to-shape (ATS) on an Elekta Unity MR-Linac. Assessing the dosimetric impact of daily online adaptations: (1) comparing dose between daily adapted (MR-adapted) and non-adapted (MR-guided) plans, by quantifying dose differences relative to reference plans (by 2 and 5%) and evaluating adaptation frequency; (2) performing intra-fraction dose evaluations. Dose metrics for targets and organs at risk (OARs) were evaluated following EMBRACE II guidelines.

**Results:**

MR-adapted plans improved target coverage or reduced OAR dose in 82–100% of fractions at a 2% dose deviation (and in 25–84% at a 5% deviation), compared to MR-guided plans. Dose reductions for OARs ranged from 2 to 8% for D0.1%, 4.77–16.70% for V4000cGy and 2.10–14.00% for V3000cGy. Intra-fraction analysis showed that the difference between daily planned and delivered doses in all target structures was not clinically significant, ranging from 0.08 to 2.20%, except two fractions that experienced higher deviations (5%) in ITV45. Treatment was well-tolerated, with no Grade 2 or 3 toxicities reported.

**Interpretation:**

MR-guided plans required adaptation in 25–100% of the fractions when compared to MR-adapted plans. MR-adapted plans demonstrated enhanced target dose consistency and reduced OAR dose for all patients, highlighting the benefits of daily adaptation. Despite longer treatment times, dose accuracy was preserved. Toxicity results for MRgART in CC appear promising.

## Introduction

The use of image-guided radiotherapy (IGRT) has significantly improved the precision and accuracy of treatment for women undergoing radiotherapy for cervical cancer (CC) [1, 2]. However, to improve outcomes, adaptive radiotherapy (ART) strategies may be needed. In CC, the inter- and intra-fractional anatomical changes in patient anatomy pose a significant challenge in the safe application of IGRT [3]. Examples include variations in organ filling such as bladder and rectum and factors like cervix motion, tumour regression or weight changes [4, 5]. Various studies have demonstrated the quantification of motion for both the target and organ at risk (OAR) [6]. These changes occur over different time scales, which can be addressed through plan adaptation strategies [7].

Hybrid MR-Linac systems offer superior soft tissue contrast and the ability to acquire multiple intrafraction images without any radiation dose compared to CT-based cone beam systems [8]. The MR-Linac provides an online ART workflow using magnetic resonance-guidance (MRgRT). The feasibility of online magnetic resonance-guided adaptive radiotherapy (MRgART) for CC has been demonstrated, with initial experience reported for patients requiring boost or phase 2 treatment [9]. They report on the overall delivered dose, workflow and treatment time. Acute toxicity in postoperative CC patients treated with MR-Linac, as well as inter- and intrafraction bladder and rectum motion in CC patients treated with MR-Linac have been presented more recently [10–12]. These analyses lack dosimetric investigation and data on daily dose and volume changes, which are essential to justify any benefits for utilising the MR-Linac for CC.

This work quantified the dosimetric impact of daily adaptation and compared it to a non-adaptive approach for patients with CC using the MR-Linac. This work assessed the inter-fraction dosimetric differences between the two approaches and determined the necessary adaptation frequency in a non-adaptive scenario. We also evaluated the dosimetric effect of intra-fraction motion on the target and OAR due to the time the patient spends on the treatment couch. This paper reports on target coverage, OAR sparing, volume changes and overall time required for the first five patients treated with a 25-fraction regimen for locally advanced CC on an MR-Linac.

## Material and method

### Patients

This retrospective analysis included the first five patients with locally advanced CC treated on the MR Linac at our institution from January 2021 to February 2024. All patients consented to 25 fractions of radiotherapy, receiving 4500 cGy/25 fractions treated over 5 weeks, and were recruited to the MOMENTUM study (NCT04075305)[13, 14]. They underwent daily MRgART on the MR-Linac (Elekta Unity AB, Stockholm, Sweden), following the fully adaptive, adapt-to-shape (ATS) protocol. All patients were staged as FIGO IIB and a median age of 43 (range 36–70). Treatment planning followed the node-negative cervix EMBRACE II protocol [15, 16], with patients undergoing concurrent chemoradiation with weekly cisplatin infusions followed by brachytherapy.

### MR-Linac imaging protocol and Workflow

Three MR images were acquired for each treatment fraction using the Philips MR Marlin System (1.5T) (V 5.7): Fx-pre, an image before the planning process used to create a daily adapted plan; Fx-mid, a verification image to confirm the patient anatomy after planning and before treatment initiation and Fx-post, used for intra-fraction evaluation post-treatment. Patients followed a bladder preparation protocol involving bladder emptying followed by drinking 300 ml of water immediately before positioning on the treatment couch. The protocol was later adjusted based on individual’s hydration status. A low-fibre diet was also advised.

The MRI scan protocol for all sessions included a T2-weighted (T2w) 2 min MRI using a Turbo Spin Echo (TSE) sequence: with a field of view (FOV) of 360 × 480 × 300 mm, and voxel size of 1.5 × 1.5 × 2.00 mm. The echo time (TE) was 183 ms, the repetition time (TR) was 1,400 m and the flip angle (FA) was 90°. The standard treatment workflow is detailed in Supplementary Figure 1. For each fraction, radiographers reported the time taken for each step of the treatment delivery. In addition, the total time between each image acquisition was recorded retrospectively, enabling a detailed analysis of treatment duration.

### Target and OAR delineation

For all reference plans (pMRs), an expert radiation oncologist in CC delineated all clinical target volumes: the high-risk primary tumour (CTV-T-HR), the low-risk primary tumour (CTV-T-LR) and the elective nodal CTV (CTV-E). All delineations followed the EMBRACE II study protocol [15, 16].

#### Inter-fraction

For Fx-pre images, a daily deformable registration (DIR) was performed using pMR. Target structures were reviewed and edited by a clinician. OAR includes the rectum, bowel and sigmoid. The bladder was rigidly propagated to account for bladder filling. Due to the treatment duration, patients are positioned before full bladder filling. To account for expected filling at mid-treatment, a rigid registration of the bladder contour was based on a full bladder on the treatment planning to reflect the anticipated filling.

#### Intra-fraction

For the intra-fraction imaging, both Fx-mid and Fx-post were contoured offline using the Monaco treatment planning system (V5.51.11). Fx-pre’s images were deformably registered to Fx-(mid and post) images for contour propagation. Experienced MR Linac radiographers (AA and CN) delineated and peer-reviewed the OARs, including the bladder, rectum, sigmoid and bowel. For each Fx-pre image, the bladder’s actual contour has been included to facilitate accurate intrafraction evaluation (Supplementary Figure 2).

### Plan generation

**Figure 1:**
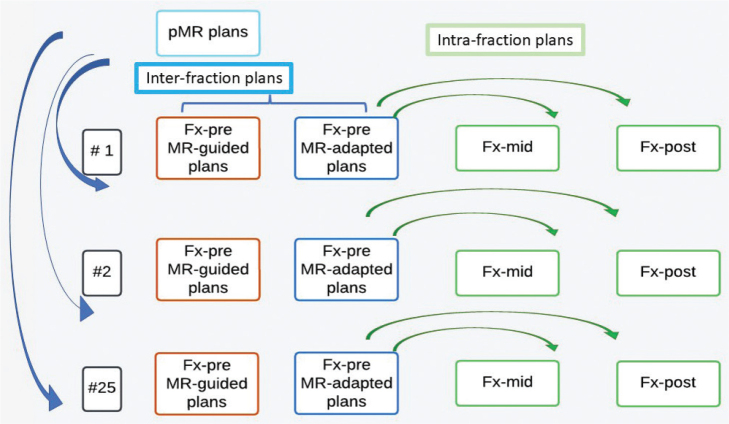
Plan generation workflow. Blue represents the inter-fraction workflow, and green represents the intra-fraction workflow. Inter-fraction: MR-adapted plans are the daily planning which is retrospectively analysed. MR-guided plans are copied from pMR to each Fx-pre to simulate inter-fraction on non-adaptive machines. Intra-fraction: MR-adapted plans are copied from Fx-pre to Fx-mid and Fx-post to estimate intra-fraction motion.

Figure 1 summarises the processes for both inter-fraction and intra-fraction plans.

#### Inter-fraction

Fx-pre (MR-adapted plans) were retrospectively analysed. In addition, MR-guided plans were retrospectively created to simulate a non-adaptive approach. This was done by transferring the plans from the pMR to each Fx-pre (MR-guided plans) using the ATS (adapt segment option, which morphs the segments to account for couch move) and recalculating the dose.

#### Intra-fraction

For intra-fraction assessment, the Fx-pre plan was transferred to the Fx-mid and Fx-post images using the ATS (original segment option, which copies the segment from image to image), and the dose was recalculated.

### Analysis

The dose metrics and structures included in the analysis were the clinical metrics for cervix treatments planned according to the EMBRACE II guidelines for node-negative cases [15, 16] (Supplementary Table 1). In addition, V3000 cGy and V4000 cGy for the sigmoid were reported as recommended by Jadon et al. [17].

#### Inter-fraction

Inter-fraction analysis involved comparing and calculating: (1) the percentage difference in median dose between the MR-guided plan and the pMR plan for each fraction, followed by taking the mean of these percentage differences across all fractions. (2) The average volume changes for target structures from Fx-pre images to pMR. The following dose metrics were considered: D0.1% for OARs and V4275 cGy for target volumes. We considered deviations on at least one structure greater than 2 or 5% as warranting adaptation, and 5% was deemed clinically meaningful. The total adaptations needed were calculated based on dose deviations in targets or one or more OAR.

#### Intra-fraction

Intra-fraction analysis assessed the percentage difference in median dose using an approach similar to the inter-fraction analysis, but comparing the dose between Fx-pre and Fx-mid, and between Fx-mid and Fx-post. Dose differences of more than 2 and 5% were considered clinically meaningful for maintaining high-quality radiotherapy delivery, using the same dose metrics as described earlier in the text [18, 19].

### Statistical analysis

#### Inter-fraction

For inter-fraction analysis, Wilcoxon Rank Sum tests were conducted to compare the MR-guided plan with the MR-adapted plan for each patient at each fraction.

#### Intra-fraction

Intra-fraction analysis involved performing a one-way ANOVA, a non-parametric (Kruskal–Wallis test) for multiple comparisons between Fx-(pre to mid) and Fx-(pre to post). Statistical significance was defined at *p* < 0.05. All statistical analyses, graphs and bar charts were generated using GraphPad Prism (version 10.2.2).

### Assessment of acute toxicity

Acute toxicity was evaluated at baseline, 6 weeks, 3 months, 6 months, 12 months and 24 months using the Common Terminology Criteria for Adverse Events (CTCAE) version 5. Assessments were based on Patient-Reported Outcome Measures (PROMs) collected by a clinical research nurse during routine follow-up visits (FU).

## Results

Of a total of 125 fractions, 119 were delivered on the MR-Linac, and the remaining fractions were treated on a conventional Linac with a non-ART plan due to machine breakdown. Median times between images for each patient are reported in Table 1. The number of images used for each patient is shown in Supplementary Table 2. The median treatment time from patient entering the treatment room to completion of treatment was 65 min (45–96). The timings of each workflow step are detailed in Supplementary Figure 3. Planning and plan checks took the longest time; median time was 19 min (12–26), followed by contouring time with a median of 15 min (9–20).

**Table 1:** Treatment time between Fx-pre to Fx-mid (adaptation time), from Fx-mid to Fx-post (treatment time) and from Fx-pre to Fx-post (total session time).

Patients	Δ Time Fx-pre and Fx-mid	Δ Time Fx-mid and Fx-post	Δ Time Fx-pre and Fx-post
1	43.5 min (range: 23–117)	13 min (range: 12–39)	57 min (range: 52–78)
2	41 min (range: 34–75)	No Fx-post	No Fx-post
3	38 min (range: 30–58)	14 min (range: 9–23)	52 min (range: 42–67)
4	38 min (range: 29–44)	15 min (range: 13–18)	50 min (range: 48–59)
5	22 min (range: 13–32)	10 min (range: 9–13)	49 min (range: 38–63)

### Inter-fraction

#### Target volume changes

Table 2 details the average weekly reduction in volume for target structures per patient. For CTV-T-LR, patients 1–4 experienced considerable reductions from baseline to week 5, with patient 2 showing the largest decrease (–72.44 cm^3^). For ITV45, the same patients all exhibited reductions in volume, with patient 4 having the largest decrease (–49.50 cm^3^). In contrast, patient 5 showed an increase in CTV-T-LR of 22.10 cm^3^ and ITV45 of 47.80 cm^3^. This was likely due to the presence of multiple large cysts changing position during treatment.

**Table 2:** Baseline and mean weekly volume changes in cm^3^ for CTV-T-LR and ITV45 structures across five patients, along with the overall target reduction from baseline to Week 5.

Structure	Patients	Baseline	Week 1	Week 2	Week 3	Week 4	Week 5	Overall target reduction
CTV-T-LR	1	80.42	85.8	80.68	76.53	68.97	55.99	–24.43
	2	209.735	167.5	158.5	158.4	146.7	137.3	–72.44
	3	84.49	81.72	78.6	79.84	77.63	72.31	–12.18
	4	113	113.7	109.5	97.95	99.33	100.8	–12.2
	5	273.7	288.7	298.5	286.5	294.5	295.8	22.1
ITV45	1	655.8	686	678.1	653.1	633.7	609.9	–45.9
	2	813.9	851.9	783.4	799.6	777	785.1	–28.8
	3	756.2	731.2	734	741.2	733	727.9	–28.3
	4	643.8	632.4	635.2	601.3	613.2	594.3	–49.5
	5	864.2	919.8	912.8	902.3	912.8	912	47.8

#### Dose comparison of MR-adapted plans versus MR-guided plans for inter-fraction motion

##### Target

**Figure 2:**
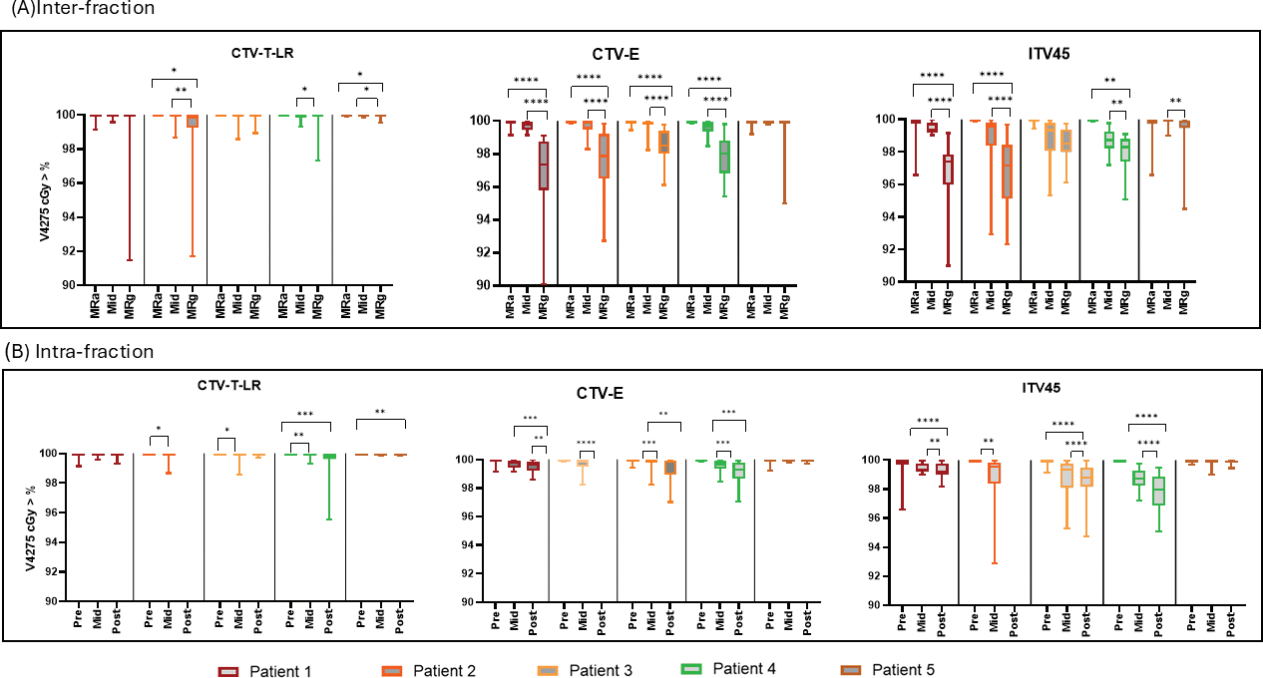
Each graph is a box plot comparison of CTV-T-LR, CTV-E and ITV45 for all patients. (A) Comparison between MR-adapted (MRa) and MR-guided (MRg) plans, and between Fx-mid plans and MR-guided plans. (B) Changes in the dose to target structures between image intervals. The plots show the median (minimum and maximum) of the dose. Each colour represents a different patient. The Y-axis displays the coverage percentage, and the X-axis shows the plans or images for each patient. Statistical significance of the *p*-values is indicated by * = *p* < 0.05, ** = *p* < 0.01, *** = *p* < 0.001 and **** = *p* < 0.0001. Abbreviations: Pre = Fx-pre, Mid = Fx-mid, Post = Fx-post.

Adaptation did not significantly improve dose coverage for CTV-T-LR in patients 1, 3 and 4 (see Figure 2A). However, there was a small but significant improvement for patients 2 (*p* = 0.0396) and 5 (*p* = 0.0352). Although the change was modest, it reduced dose variability in MR-adapted plans compared to MR-guided plans across all patients.

Significant underdosing was observed with MR-guided plans compared to MR-adapted plans for CTV-E and ITV45 in all patients except patient 5, who showed no significant change (Figure 2A). CTV-E dose coverage improved with adaptation by 3.00, 2.50, 2.13 and 1.81% for patients 1 to 4, respectively (all *p* < 0.0001). For ITV45, dose coverage improved with adaptation by 3.15, 3.08, 1.33 and 1.93% for patients 1 to 4, respectively (all *p* < 0.0001). Despite the potential for dose degradation due to adaptation time (Table 1, Supplementary Figure 3), Figure 2A shows that Fx-mid plans consistently outperformed MR-guided plans across all target structures and patients, demonstrating the true benefit of adaptation. In patients 1 and 5, Fx-mid plans achieved better dose coverage than MR-adapted plans.

##### Organs at risk

Most of the OAR dose metrics showed statistically significant reductions with MR-adapted plans compared to MR-guided (see Supplementary Table 3). The D0.1% for bladder showed reductions ranging from 2.30 to 8.00%, sigmoid from 2.00 to 4.50% and bowel ranged from 2.00 to 4.50%. Bladder V4000 cGy reductions ranged from 4.77 to 11.70%, and V3000 cGy from 6.90 to 7.30% (patients 1, 3 and 5). Bowel V4000 cGy reductions ranged from 6.50 to 16.70% (patients 1, 3 and 4), and bowel V3000 cGy was significantly reduced in patients 1, 2 and 5, ranging from 2.10 to 14.00%. The sigmoid consistently showed dose reductions across all dose metrics (Supplementary Table 3).

However, there are a few cases where doses increased with adaptation (see Supplementary Table 3). Patient 4 significantly increased bladder V4000 cGy (6.01%, *p* = 0.0004). Bowel V4000 cGy for patient 5 increased significantly by 3.70% (*p* < 0.0001). Rectum D0.1% increased by 2.10% in patient 1 (*p* = 0.0012). The rectum increased in either V3000 or V4000 cGy with adaptation: significant increases in V4000 cGy were observed in patients 1 and 5 (3.85 and 12.35%), while V3000 cGy increased significantly in patients 4 and 5 (3.30 and 5.80%), respectively.

#### Intra-fraction

##### Target

Figure 2B illustrates the intra-fraction dose variability among patients for target structures. Overall, the dose coverage remained high and consistently within CTV-T-LR, but increased dose variation was observed in CTV-E and ITV45. For ITV45, statistically significant dose reductions were observed for all patients except patient 5 at all image intervals available (all *p* < 0.01). Patient 5 consistently maintained ITV45 coverage across images with minimal, non-significant dose deviation, attributed to less time spent on the bed (Fx-pre to mid: *p* > 0.99, 22 min; Fx-pre to post: *p* = 0.0848, 48 min). Despite statistically significant findings, the mean dose reduction from Fx-pre to Fx-mid for all patients was not considered clinically significant, ranging from 0.18 to 1.29%, and from Fx-pre to Fx-post, ranging from 0.08 to 2.20%, with patients 3 and 4 experiencing higher dose deviations. The workflow duration ranged from 22–43.5 min from Fx-pre to Fx-mid and 49–57 min from Fx-pre to Fx-post.

##### Organs at risk

Actual bladder filling in the Fx-pre image increased from Fx-pre to mid by a median of 76 cm³ (range: 6.40–485.97 cm³) and from Fx-pre to post by a median of 157 cm³ (range: 20–553.23 cm³). Volume remained consistent at all imaging intervals for the rectum and sigmoid across all patients. Considerable reductions were observed in the bowel volume for patients 1, 3 and 4 at all intervals expected due to the increase in bladder volume during the session, displacing the bowel.

Supplementary Table 4 presents the percentage dose differences and corresponding *p*-values for all relevant DVH metrics and OARs between the estimated delivered dose (Fx-pre to mid) and the post-treatment dose (Fx-pre to post).

For D0.1%, both bladder and bowel exhibited a statistically significant but not clinically significant dose increase between image intervals ranging from (0.20 to 1.20%). Sigmoid and rectum D0.1% was consistent for all patients at all intervals except for a significant decrease in dose to the rectum was noted for patient 4 Fx-pre to post by 0.70% *p* < 0.0001.

For V4000 cGy, mixed results were found in the bladder, where patient 1 experienced a significant increase between intervals and patient 4 showed a decrease in V4000 cGy. Overall, V4000 cGy showed a reduction in the rectum (0.42 to 11.04%), bowel (3.93 to 54.40%) and sigmoid (0.66 to 15.35%) between image intervals.

For V3000 cGy, the bladder dose decreased for patients 3 and 5, ranging from 9.28 to 31.38%. No significant dose changes were found for the rectum and sigmoid. The irradiated volume reduced in the bowel ranged between 1.70 and 34.97%.

### Dose deviation and number of fractions requiring adaptation

**Figure 3:**
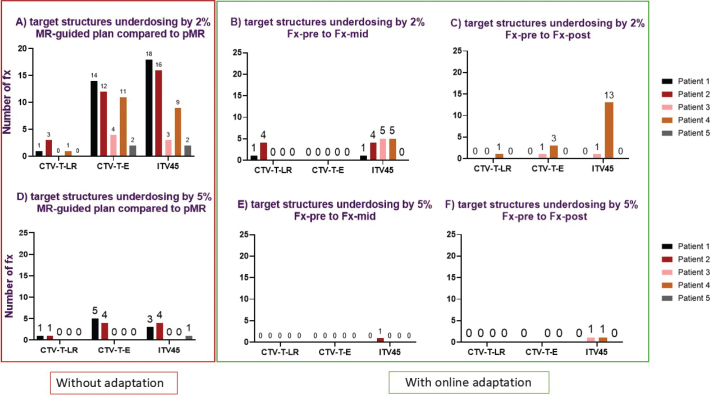
The number of fractions with target underdosing based on a 2% (top row) and 5% (bottom row) threshold for target coverage variation (V4275 cGy). The red square (A/D) highlights the number of fractions with dose deviations of 2 and 5%, indicating the required fractions for adaptation by comparing MR-guided plans to the reference plan (pMR). The green square shows dose deviation in each image interval during an online adaptive session. (B/E): display dose deviations of 2 and 5%, comparing Fx-pre to Fx-mid, while (C/F) show deviations of 2 and 5%, comparing Fx-pre to Fx-post. Different colours represent different patients.

**Figure 4:**
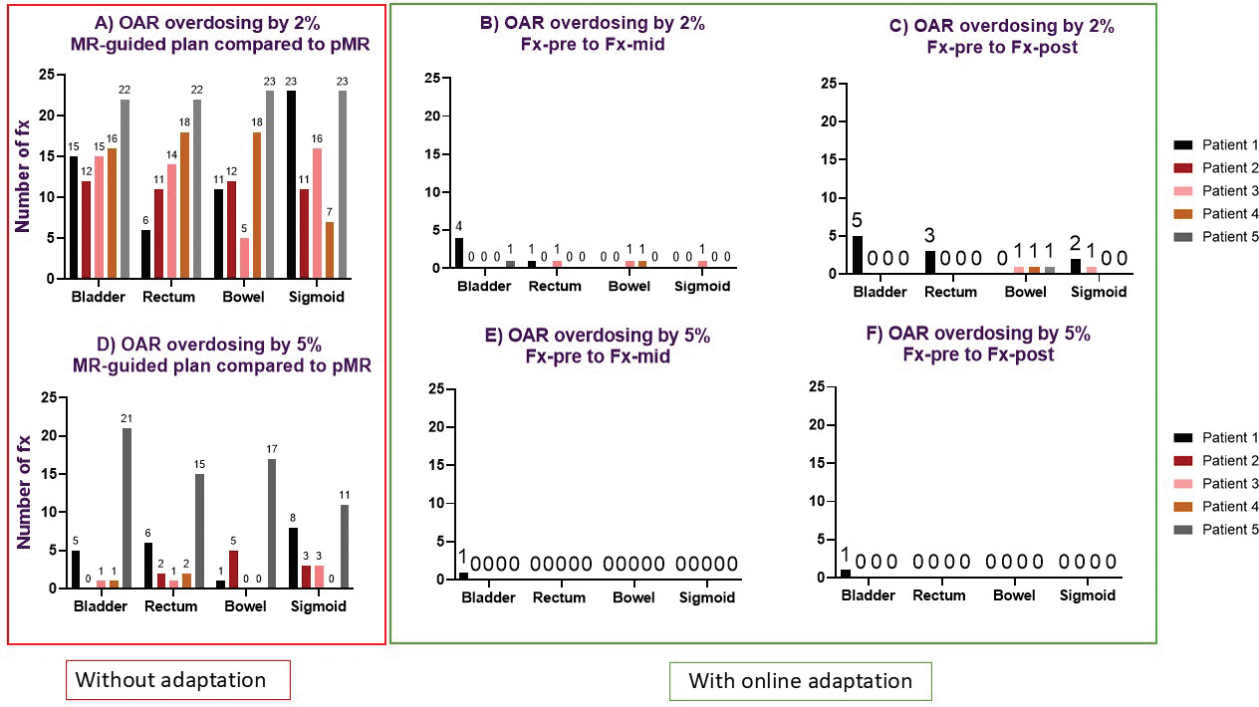
The number of fractions with dose variation based on a 2% (top row) and 5% (bottom row) threshold for OAR (D0.1%). The red square (A/D) highlights the number of fractions with dose deviations of 2 and 5%, indicating the required fractions for adaptation by comparing MR-guided plans to the reference plan (pMR). The green square shows dose deviation in each image interval during an online adaptive session. (B/E) display dose deviations of 2 and 5%, comparing Fx-pre to Fx-mid, while (C/F) show deviations of 2 and 5%, comparing Fx-pre to Fx-post. Different colours represent different patients.

**Figure 5:**
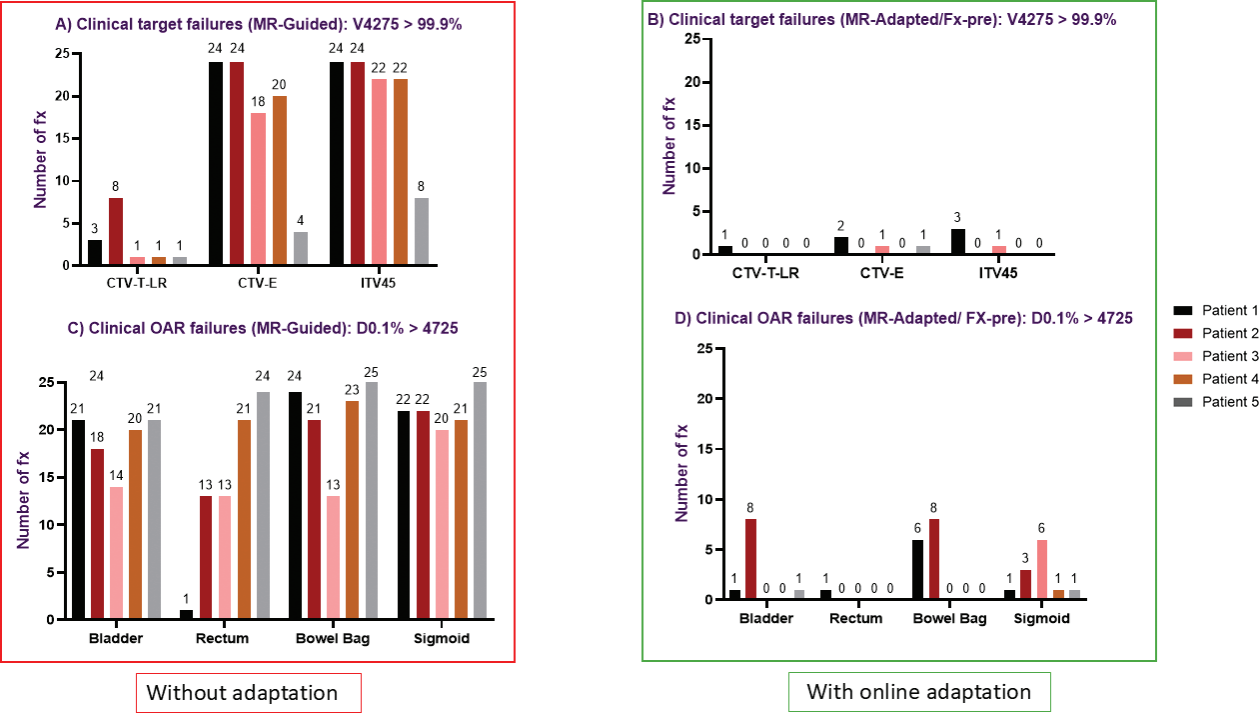
Comparison of clinical target and OAR dose constraint failures with online adaptation (MR-adapted/ Fx-pre) and without online adaptation (MR-guided). (A/B) show fractions failing target coverage (V4275 > 99.9%) for CTV-T-LR, CTV-E and ITV45. (C/D) show fractions failing OAR constraints (D0.1% > 4725 cGy) for bladder, rectum, bowel bag and sigmoid.

For the MR-adapted plan, the inter-fraction dose deviation (between pMR and Fx-pre) was less than 2% for all patients and structures. Figures 3 and 4 detail the number of fractions underdosing to the target and overdosing to OARs if adaptation was not used. From Figures 3 and 4, it is evident that the need for adaptation was primarily driven by overdosing on OARs rather than target underdosing. The number of fractions requiring adaptation per patient with a dose deviation of 2% is as follows: Patient 1: 24/24 (100%), Patient 2: 21/24 (88%), Patient 3: 18/22 (82%), Patient 4: 24/24 (100%) and patient 5: 22/25 (88%). The number of fractions requiring adaptation per patient with a dose deviation of 5% is as follows: Patient 1: 14/24 (58%), Patient 2: 12/24 (50%), Patient 3: 10/22 (45%), Patient 4: 6/24 (25%) and Patient 5: 21/25 (84%). Figure 5 demonstrates that without adaptation, target coverage and OAR sparing were compromised in most fractions across all patients. The number of clinical failures in the MR-guided plans aligns closely with the 2% dose variation threshold (Figures 3, 4), with a slightly reduced number of fractions requiring adaptation.

Figures 3 and 4 demonstrate that the frequency of target underdosing events and OAR overdosing notably decreases when online adaptation is employed, particularly for dose deviations of 5%. The number of fractions exhibiting a 5% underdose was modest in all cases, with only two fractions experiencing underdosing for ITV45 from Fx-pre to post and only one fraction for the shorter interval (Fx-pre to mid) (Figure 3F). Additionally, there was only one instance of bladder overdosing by 5% (Figure 4F). This suggests that shorter treatment times can improve dosimetric outcomes.

**Figure 6:**
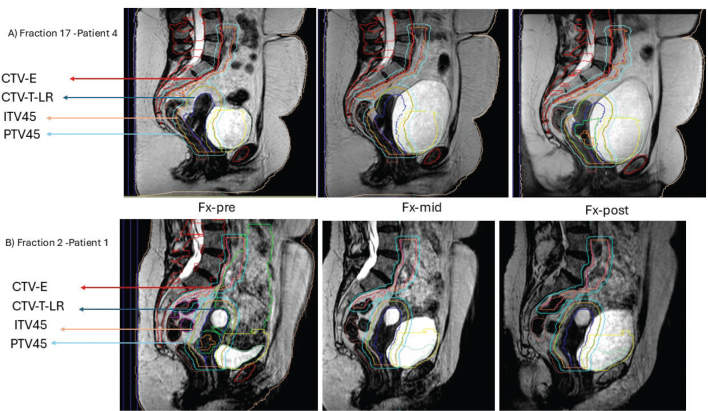
Impact of bladder filling on ITV45 coverage. (A) Patient began treatment with a full bladder (218.34 cm³), close to the planned MR (pMR) bladder volume of 202.55 cm³. By the Fx-mid image, the bladder volume increased to 491.18 cm³, pushing the CTV-T-LR (navy contour) posteriorly. During the Fx-post image, the bladder volume reached 528.27 cm³, causing the ITV45 (orange contour) to inadequately cover the entire CTV-T-LR. (B) The patient started treatment with a half-full bladder, maintaining sufficient ITV45 coverage at all imaging intervals.

A 2% underdose for ITV45 in 13 fractions for patient 4 (Figure 3C) was noted. These fractions involved an extremely full bladder (volume >500 cm³), which pushed the target posteriorly (see Figure 6).

#### Reporting of acute toxicity

All patients tolerated the treatment well. Patients 1–4 completed FU evaluations at 6 weeks, 3 months, 6 months, 12 months and 24 months. Patient 5, who recently completed treatment, has undergone 3 weeks and 3 months of FU. Fatigue grade 1 (G1) was a common finding at all time points. At the 6-week follow-up, three patients reported no toxicities, and at the 24-month follow-up, no toxicities were reported for patients 1 to 4. Patients developed mild acute toxicities (G1) between 6 weeks and 12 months FU which included G1 diarrhoea, urinary urgency, urinary frequency, vaginal dryness, vaginal discharge, rectal pain and cystitis. No grade 2 or grade 3 toxicities were reported (Supplementary Table 5).

## Discussion

To our knowledge this is the first paper to evaluate and compare the delivered dose between MR-guided daily adaptation (ATS) and non-adaptation in CC patients receiving 25 fractions. Our main finding is that MR-adapted plans, with daily dose monitoring, improved either target coverage or reduced OAR dose in 82–100% of total fractions when evaluating a 2% dose deviation and in 25–84% of total fractions when evaluating a 5% dose deviation, compared to MR-guided plans. This highlights the fact that all patients benefited from adaptation; however, the number of daily adaptations required over a total treatment course appears to be patient-dependent and requires a larger cohort to better understand and quantify this variability.

The ITV45 target reduction in volume ranged from 28.8 to 45.9 cm³, beginning as early as week 2 (Table 2), suggesting that significant reductions may necessitate daily adaptation. With MR-adapted plans, patients experienced significant dose reductions in the bladder, sigmoid and bowel and this would suggest a reduction in patient toxicities (Supplementary Table 3). However, the dose to the rectum did not significantly reduce, particularly the V4000 cGy, compared to MR-guided plans. This may be because the conformity of ATS planning to improve target coverage can impact the dose received by some OARs. However, when evaluating the delivered rectum dose on the Fx-mid image, we observed a reduction in V4000 cGy, ranging from 0.42 to 11.04%.

The intra-fraction analysis underscored the fact that while two patients experienced ITV under-dosing (5% in two fractions [Figure 3F]), most patients maintained stable dose coverage throughout the treatment intervals. This indicates that the ITV margin is sufficient to compensate for movement during long treatment sessions and that the benefit of daily adaptation is not affected by intra-fraction motion. For OAR (V3000 cGy and V4000 cGy) between Fx-pre and Fx-mid, the result was optimised by adaption for most patients. The observed variations in dose and time, particularly between Fx-pre and Fx-post, suggest that shorter intervals can improve the accuracy of the delivered dose.

Previous research suggests that slight variations in radiotherapy dose delivery may have a significant impact on tumour control and the risks of radiation toxicity [20, 21]. A 2% change in the delivered dose can arise from various sources, including daily dose fluctuations, and can result in up to a 10% variation of the tumour control probability (TCP) for steep dose–response curves [21]. In this study, we observed greater consistency in 2% dose deviations in the non-adaptive scenarios. We suggest that a daily dose variation of 2% may impact both tumour control and toxicity risk, warranting further investigation to enhance the effectiveness of daily adaptation.

Dose deviations appeared randomly across fractions without a clear time trend, largely due to variations in bladder filling during imaging intervals. This variability in bladder filling impacts target dose and OAR exposure, underscoring the importance of daily adaptation. In our study, bladder filling variability appears to be the main reason for applying daily adaptation, as highlighted in previous studies [22–24]. This experience treating CC patients on the MR-Linac suggests that preparing patients with either an empty or half-full bladder at the first image of each fraction to allow natural filling during planning may improve dose accuracy (see Figure 6).

The extended treatment duration is a key concern when treating CC patients on MR-Linac with ATS. In our study, the time between Fx-pre and Fx-post images ranged from 49 to 57 min (Table 1), with a total median treatment time (entry to exit) of 65 min (45–96 min). This is notably longer than previous MR-Linac studies for CC, which reported times of 32 min (29–35 min) [11] and 42 min (30–65 min) [25]. In another cohort, the median in-room time was 35 min [10], the median room occupancy times was 45 min (38–50 min) [12]. The shorter treatment times in these studies can be attributed to the fact that ATS was not used in all fractions.

Treatment time is a major logistical barrier in radiotherapy departments and affects patient comfort. Recent literature shows that AI-based contouring tools could be useful online for recontouring for adaptive MR-guided radiotherapy [26, 27]. AI-Rad Companion Organs RT™ demonstrated a good performance, with 80% clinically acceptable contours in this preliminary study for various anatomical sites and led to time savings [26]. However challenges may present, integrating this into an MR-linac system.

Our study was not designed to evaluate treatment-related toxicity; however, with MR-linac ATS adaptation, we observed a low incidence of toxicity. This suggests that daily online adaptation may further reduce the dose to OARs, potentially lowering the risk of such toxicities [28]. Future clinical trials are required for validation.

The main limitation of this study is its statistical power as a result of the small number of patients, which may restrict the detection of meaningful differences or effects. Interpreting statistical significance in small cohorts requires caution. While statistical significance indicates the reliability of results, clinical significance relates to their practical impact [29, 30]. In this study, we use a 5% threshold to indicate clinical significance between planned and delivered doses and evaluate patients’ toxicity symptoms to ascertain the true benefits of MRgART.

A recent review has highlighted a data gap in MRgART and noted that the library of plan adaptation (LOP) is the most used clinical approach [31]. Further studies are needed to compare MRgART, particularly ATS, with LOP to better assess the need for daily online adaptation.

## Conclusion

MR-adaptive plans reduced target dose variability and reduced OAR exposure compared to MR-guided alone. The dosimetric results improved in 25–100% of fractions compared to MR-guided plans, highlighting the benefits of daily adaptation. Despite longer treatment times, intra-fraction motion did not diminish the benefit of online adaptation. Shortening treatment times could further enhance accuracy by reducing bladder filling variation. Acute and late toxicity results are promising for MRgART in CC.

## Author contributions

AA and CLE were guarantors of the integrity of the entire study. All authors contributed to the study’s conception and design. AA conducted the literature review search, contouring, dosimetry planning data analysis, and manuscript preparation, which was subsequently reviewed by CLE, MAZ, PH, RC and CN. All authors contributed to the manuscript editing, RC reviewed dosimetry plans and result analysis and CN reviewed the contours.

## Supplementary Material



## Data Availability

The data used in this study were obtained from [MOMENTUM study (NCT04075305)]. Access to these data can be requested from MOMENTUM@lygature.org.
