# Supplementary material for: Quantifying the dosimetric impact of online daily adaptation for MR-guided RT in cervical cancer
Source: Acta Oncol. 2025 May 19;64:42898. doi: 10.2340/1651-226X.2025.42898 (PMC12108135; doi:10.2340/1651-226X.2025.42898)
Supplement: Supplementary file 1 [file AO-64-42898-s1.pdf]

Supplementary material has been published as submitted. It has not been copyedited, or typeset by Acta Oncologica

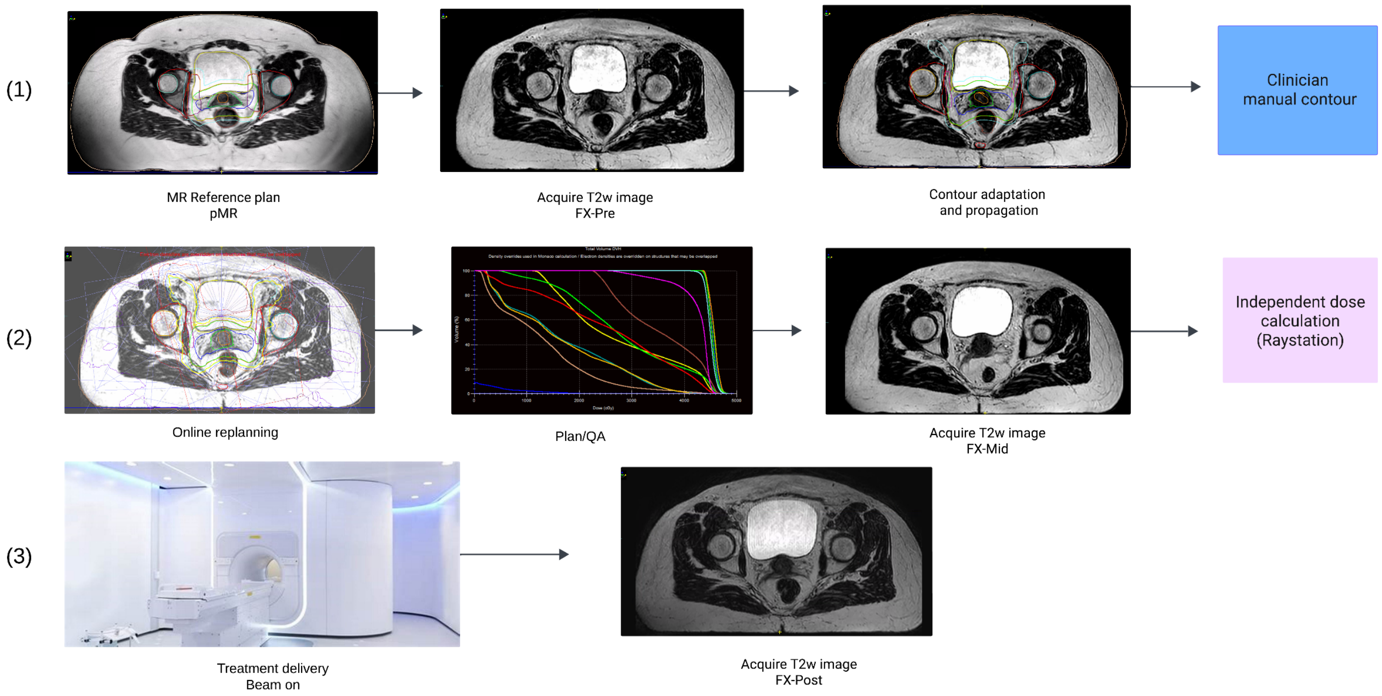

Figure 1: **MR-Linac ATS workflow.** Fx-pre is the ATS workflow's starting point (see Figure 1), known as the MR-adapted plan. The ATS workflow involves daily deformable image registration (DIR) from the planning reference MR (pMR) image to propagate contours onto the daily Fx-pre, followed by a clinician's manual editing of the target and OARs, and a new optimised plan to accommodate daily anatomical variations. Following an initial quality assurance check (QA) using Monaco's treatment planning system (TPS) (V5.51.11), a Fx-mid image was acquired to confirm the patient's position. A second independent QA assessment is performed using Raystation TPS (Raystation 11B, V.12) (RaySearch Laboratories, Stockholm, Sweden). Finally, a Fx-post image was acquired two minutes before beam-off to minimise patient couch time

1- Bladder Contour

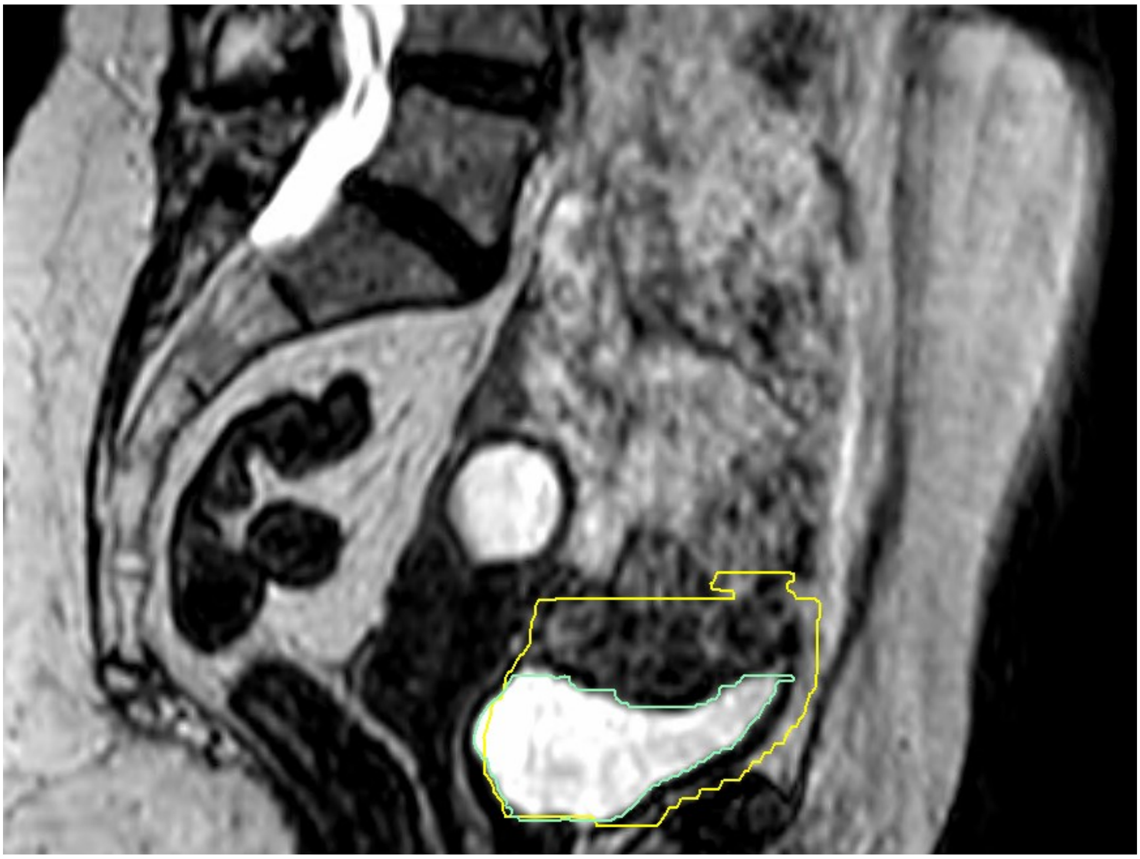

Figure 1: An example of a propagated rigid bladder contour shown in yellow, while the actual bladder filling is delineated in green.

Category	Structure	Constraint	Value
Hard Constraints	Target Structures		
	CTV-E	V4275 cGy	> 99.9%
	CTV-T-LR	V4275 cGy	> 99.9%
	ITV-45	V4275 cGy	> 99.9%
	Organ at Risk		
	Bladder	D0.1%	< 4725 cGy
	Rectum		
	Bowel		
	Sigmoid		
Soft Constraints	Bladder	V4000 cGy	< 60% (+40%)
		V3000 cGy	< 80% (+20%)
	Bowel	V4000 cGy	< 250 cm^3
		V3000 cGy	< 500 cm^3
	Rectum	V4000 cGy	< 75% (+25%)
		V3000 cGy	< 95% (+5%)
	Sigmoid	V4000 cGy	< 100 cm^3
		V3000 cGy	< 90 cm^3

Table 1: Clinical goals (in cGy) for cervix treatments planned to the EMBRACE II guidelines for node negative patients. Hard constraints are crucial for treatment planning, while soft constraints are more flexible and adjustable based on the importance of planning

Patient	Fx-pre	Fx-mid	Fx-post
**1**	24	24	24
**2**	24	24	0
**3**	22	22	11
**4**	24	24	22
**5**	25	25	25

Table 2: Number of interaction and intrafraction images available per patient

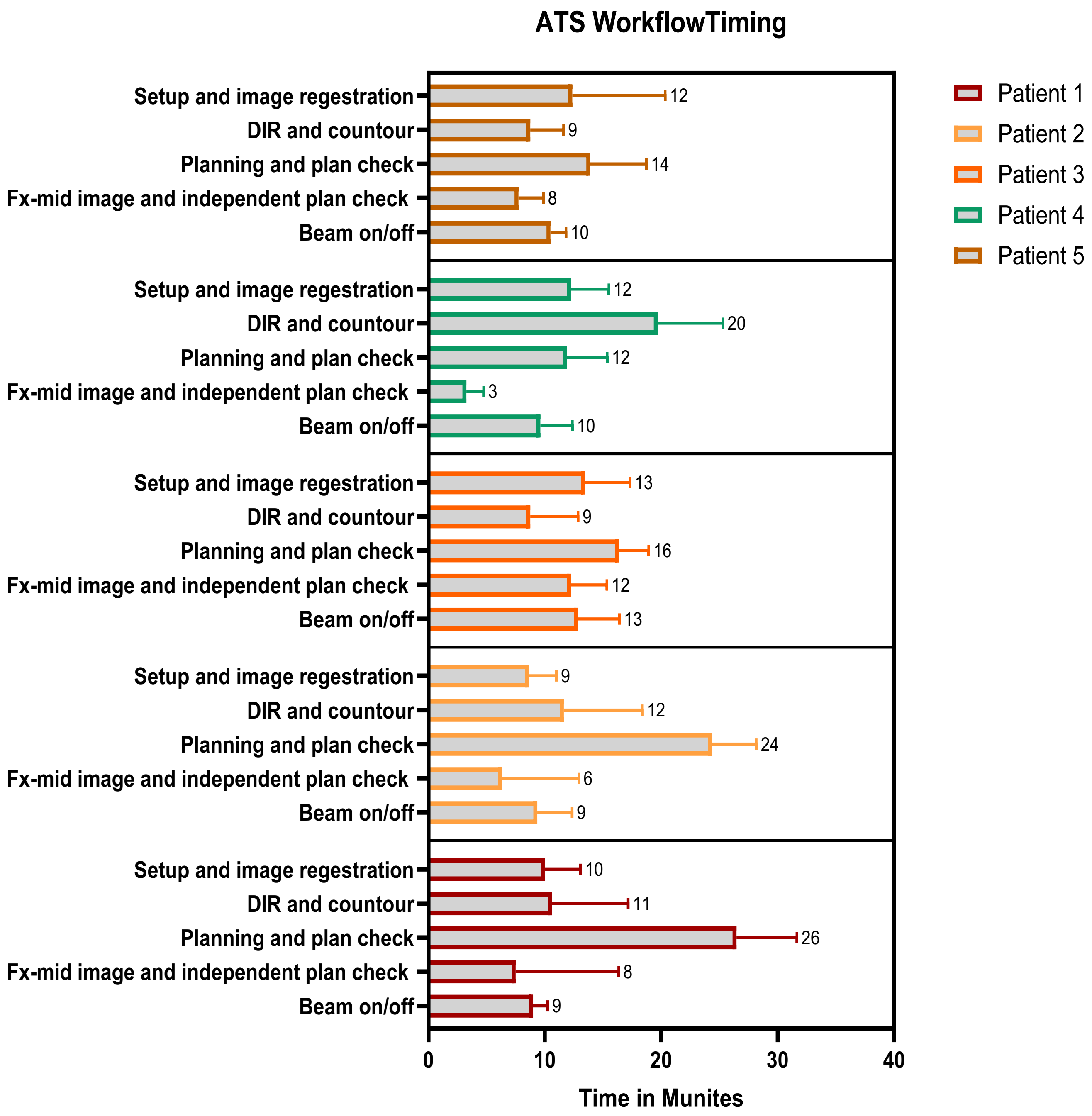

Figure 3: Mean error bar explaining ATS workflow timing for all patients. Planning and contouring are the most time-consuming steps. DIR: deformable registration

Table 3: OAR dose changes with MR-adaptive plans compared to MR-guided plans. Increases (↑) and decreases (↓) in dose are indicated, with "% Diff" representing the percentage difference. Grey highlights insignificant p-values.

OAR	Dose metric	Patients	Dose ↓↓ or ↑↑ with adaptation	% Diff	P value	OAR	Dose metric	Patients	Dose ↓↓ or ↑↑ with adaptation	% Diff	P value
Bladder	D0.1%	1	↓	2.70%	p<0.0001	Bowel	D0.1%	1	↓	2.30%	p<0.0001
		2	↓	2.10%	p=0.0070			2	↓	2.70%	p<0.0001
		3	↓	2.80%	p=0.0001			3	↑	0.70%	p=0.1289
		4	↓	2.30%	p<0.0001			4	↓	3.10%	p<0.0001
		5	↓	8.00%	p<0.0001			5	↓	5.70%	p<0.0001
	V4000 cGy	1	↓	8.62%	p<0.0001		V4000 cGy	1	↓	16.70%	p<0.0001
		2	↓	0.40%	p=0.8537			2	↑	30.00%	p=0.1021
		3	↓	6.20%	P=0.0388			3	↓	0.60%	p=0.0501
		4	↑	4.77%	P=0.0004			4	↓	6.50%	p=0.0043
		5	↓	11.70%	P=0.0003			5	↑	3.70%	p<0.0001
	V3000 cGy	1	↓	6.90%	p=0.0009		V3000 cGy	1	↓	14.00%	p<0.0001
		2	↓	0.08%	p=0.627			2	↓	3.20%	p=0.0061
		3	↓	6.40%	p=0.0056			3	↓	0.60%	p=0.0748
		4	↑	6.01%	P=0.0673			4	↓	0.00%	p=0.2146
		5	↓	7.30%	p<0.0001			5	↓	2.10%	p=0.0014
Rectum	D0.1%	1	↑	2.10%	p=0.0012	Sigmoid	D0.1%	1	↓	4.40%	p<0.0001
		2	↓	1.12%	p=0.7204			2	↓	2.30%	p=0.0006
		3	↓	3.60%	p=0.0431			3	↓	2.30%	p<0.0001
		4	↓	3.10%	p<0.0001			4	↓	2.00%	p<0.0001
		5	↓	6.00%	p<0.0001			5	↓	4.50%	p<0.0001
	V4000 cGy	1	↑	12.35%	p=0.0002		V4000 cGy	1	↓	1.80%	p=0.0794
		2	↑	1.12%	p=0.7204			2	↓	5.00%	p=0.0466
		3	↓	3.60%	p=0.0431			3	↓	2.30%	p=0.00421
		4	↓	1.30%	p=0.4278			4	↓	15.00%	p=0.0001
		5	↑	3.85%	p=0.0002			5	↓	0.00%	p=0.8344
	V3000 cGy	1	↑	3.40%	p=0.0727		V3000 cGy	1	↓	0.70%	p=0.992
		2	↓	1.78%	p=0.01			2	↓	5.00%	p<0.0001
		3	↑	0.60%	p=0.8596			3	↓	0.60%	p=0.0917
		4	↑	5.80%	p=0.0240			4	↓	0.00%	p=0.134
		5	↑	3.30%	p=0.014			5	↓	0.00%	p=0.8344

Table 4: OAR dose changes during MR-Adaptive Sessions: Fx-Pre to Fx-Mid and Fx-Pre to Fx-Post. Significant dose increases (↑), reductions (↓), and non-significant results (↓/↑) are indicated. "Diff" represents the percentage difference, grey highlights significant p-values.

OAR	Dose metric	Patients	Dose changes from Fx-pre to Fx-mid	Dose changes from Fx-pre to Fx-post	OAR	Dose metric	Patients	Dose changes from Fx-pre to Fx-mid	Dose changes from Fx-pre to Fx-post
Bladder	D0.1%	1	↑ 0.80% p=0.0067	↑1.00% p=0.0033	Bowel	D0.1%	1	↑ 0.20% p>0.9999	↑ 0.20% p>0.9999
		2	↑ 0.40% p=0.1186	NA			2	↑ 0.80% p<0.0001	NA
		3	↑ 0.20% p>0.999	↑ 0.50% p=0.6631			3	↑ 0.60% p=0.0049	↑ 0.70% p=0.0151
		4	↑ 0.40% p=0.0062	↑ 0.70% p<0.0001			4	↑ 0.30% p=0.0556	↑ 0.20% p=0.0766
		5	↑ 0.80% p=0.0002	↑ 1.20% p<0.0001			5	↓ 0.30% p=0.6312	↓ 0.20% p=0.0208
	V4000 cGy	1	↑ 5.60% p=0.0248	↑ 6.5% p=0.0078		V4000 cGy	1	↓ 40.00% p=0.0077	↓54.50% p=0.0013
		2	↑ 1.86% p>0.9999	NA			2	↓ 5.24% p=0.4223	NA
		3	↓ 9.070% p=0.919	↓18.80% p=0.1106			3	↓ 5.15% p>0.9999	↓ 7.66% p=0.0528
		4	↓ 23.14% p<0.0001	↓28.61% p<0.0001			4	↓ 27.20% p=0.3085	↓ 42.95 p=0.0331
		5	↑ 2.60% p>0.9999	↑ 7.99% p=0.1871			5	↓ 3.93% p>0.9999	↓ 5.89% p=0.4602
	V3000 cGy	1	↑ 10.50% p=0.1892	↑13.00% p=0.0533		V3000 cGy	1	↓ 23.60% p=0.0724	↓ 34.97% p=0.0106
		2	↑ 0.75% p>0.9999	NA			2	↓ 2.99% p=0.6431	NA
		3	↓ 9.28% p=0.0200	↓14.95% p=0.0047			3	↓ 7.13% p>0.9999	↓ 0.22% p>0.9999
		4	↑ 0.96 % p>0.9999	↑ 0.16 % p>0.9999			4	↓ 14.45% p>0.9999	↓ 25.49% p=0.6494
		5	↓ 31.38% p<0.0001	↓ 26.99% p<0.0001			5	↓ 1.7% p>0.9999	↓ 4.40% p=0.6638
Rectum	D0.1%	1	↓ 0.35% p>0.9999	↓ 0.50% p>0.9999	Sigmoid	D0.1%	1	↑ 0.10% p=0.9124	↑ 0.30% p=0.4722
		2	↑ 0.30% p=0.1389	NA			2	↑ 0.50% p=0.0002	NA
		3	↓ 0.10% p>0.9999	↓ 0.10% p=0.8360			3	↓ 0.10% p>0.9999	↓ 0.10% p>0.9999
		4	↓ 0.50% p=1078	↓ 0.70% p<0.0001			4	↑ 0.30% p=0.4744	↑ 0.30% p=0.4041
		5	0% p>0.9999	0% p=0.2899			5	↓ 0.10% p>0.9999	↓ 0.30% p=0.3866
	V4000 cGy	1	↑ 1.78 % p>0.9999	↑1.724 % p>0.9999		V4000 cGy	1	↓ 8.87% p=0.3064	↓ 9.56% p=0.3064
		2	↓ 0.42% p=0.7955	NA			2	↓ 4.01% p=0.2638	NA
		3	↓ 5.62% p=0.0165	↓ 6.43% p=0.0376			3	↓ 8.71% p=0.3548	↓ 8.19% p=0.3565
		4	↓ 7.09% p=0.0395	↓ 11.03 p=0.0047			4	↓ 0.66% p>0.9999	↓ 1.5% p>0.9999
		5	↓ 1.69% p=0.7009	↓ 1.96% p=0.9668			5	↓ 12.80% p=0.2181	↓ 15.35% p=0.1098
	V3000 cGy	1	↑ 0.76% p>0.9999	↑ 1.94 % p>0.9999		V3000 cGy	1	↓ 9.04% p=0.3064	↓ 15.19% p=0.0167
		2	↑ 0.71% p=0.3633	NA			2	↓ 8.89% p=0.0770	NA
		3	0% p>0.9999	0% p>0.9999			3	↓ 9.18% p=0.1207	↓ 7.69% p=0.7569
		4	↓ 3.56% p=0.5903	↓ 1.96% p=0.2727			4	↓ 12.17% p=0.9013	↓ 20.10% p=0.2944
		5	↑ 0.47% p>0.9999	↑ 0.51% p>0.9999			5	↓ 13.54% p=0.2000	↓ 14.57% p=0.1356

Table 5: Toxicity evaluation based on the Common Terminology Criteria for Adverse Events (CTCAE) across multiple timepoints

	**Baseline**	**6 Weeks**	**3 Months**	**6 Months**	**12 Months**	**24 Months**
**Patient 1**	No toxicities present	No toxicities present	G1 Diarrhoea<br>G1 rectal pain	No toxicities present	G1 fatigue<br>G1 urinary urgency<br>G1 urinary frequency	No toxicities present
**Patient 2**	No toxicities present	No toxicities present	No toxicities present	G1 fatigue<br>G1 diarrhoea<br>G1 urinary frequency<br>G1 vaginal dryness<br>G1 vaginal discharge	G1 vaginal discharge	No toxicities present
**Patient 3**	G1 fatigue<br>G1 Nausea<br>G1 constipation<br>G1 urinary frequency	G1 Fatigue	G1 Fatigue<br>G1 Urinary frequency	G1 Fatigue	G1 Fatigue	No toxicities present
**Patient 4**	No toxicities present	G1 Fatigue	No toxicities present	No toxicities present	No toxicities present	No toxicities present
**Patient 5**	G1 constipation<br>G1 vaginal discharge	G1 Cystitis<br>G1 Urinary frequency	G1 Urinary frequency<br>G1 Vaginal discharged	Not due yet	Not due yet	Not due yet
